# 392. Favorable Adherence and Safety of Twice-Yearly Subcutaneous Lenacapavir for PrEP Among PURPOSE 2 Participants Who Used Substances

**DOI:** 10.1093/ofid/ofaf695.130

**Published:** 2026-01-11

**Authors:** Jesse ClarkAllison Agwu, Susan P Buchbinder, Jill Blumenthal, Nittaya Phanuphak, Joanne Batting, Michelle S Cespedes, Beatriz Grinsztejn, Javier R Lama, Karam Mounzer, Priyanka Arora, Lillian B Brown, Christoph C Carter, Sara Salerno, Renu Singh, Pamela Wong, Carlo Hojilla, Susanne Doblecki-Lewis

**Affiliations:** Johns Hopkins University School of Medicine, Baltimore, MD; San Francisco Department of Public Health, San Francisco, California; University of California San Diego, San Diego, CA; Institute of HIV Research and Innovation – Pribta Tangerine Clinic, Bangkok, Krung Thep, Thailand; Foundation for Professional Development (FPD), Ndevana Community Research Site, Ndevana, Eastern Cape, South Africa; Icahn School of Medicine at Mount Sinai, New York, New York; 15. Instituto Nacional de Infectologia Evandro Chagas, Fundação Oswaldo Cruz, Rio de Janiero, Rio de Janeiro, Brazil; Asociación Civil Impacta Salud y Educacion, Lima, Lima, Peru; Philadelphia Fight Community Health Centers, Philadelphia, Pennsylvania; Gilead Sciences, Inc., Foster City, California; Gilead Sciences, Inc., Foster City, California; Gilead Sciences, Inc., Foster City, California; Gilead Sciences, Inc., Foster City, California; Gilead Sciences, Inc., Foster City, California; Gilead Sciences, Inc., Foster City, California; Gilead Sciences, Foster City, California; University of Miami Miller School of Medicine, Miami, Florida

## Abstract

**Background:**

Cisgender men and gender-diverse individuals with a high likelihood of HIV acquisition are disproportionately affected by substance use, which can affect HIV pre-exposure prophylaxis (PrEP) adherence. Twice-yearly subcutaneous (SC) lenacapavir (LEN) showed high efficacy and safety for PrEP in PURPOSE 2 (NCT04925752). We investigated the impact of substance use on LEN adherence and safety in PURPOSE 2. As LEN is an inhibitor of CYP3A, an enzyme involved in fentanyl metabolism, we developed a physiologically based pharmacokinetic (PBPK) model to evaluate this potential drug–drug interaction.

**Figure:**
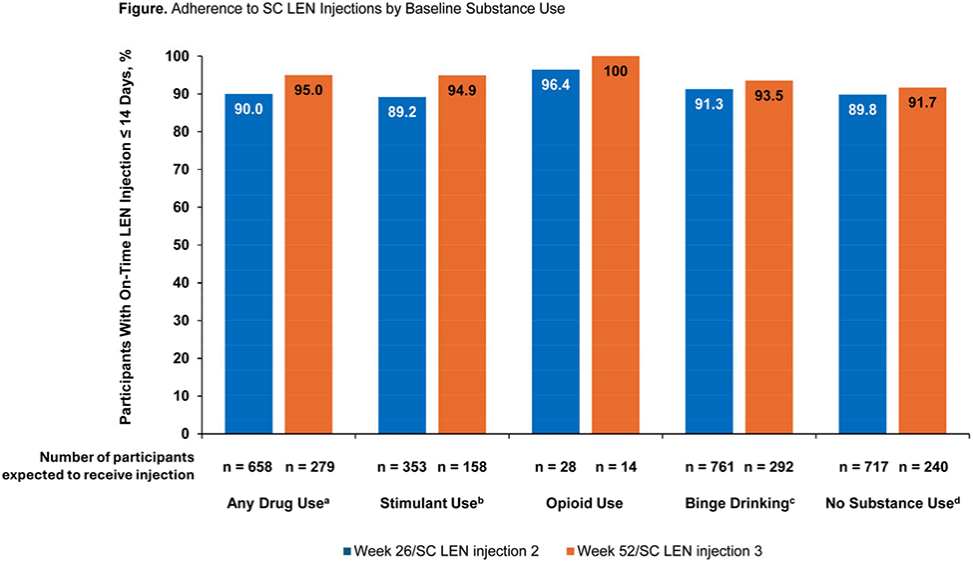

**Figure:**
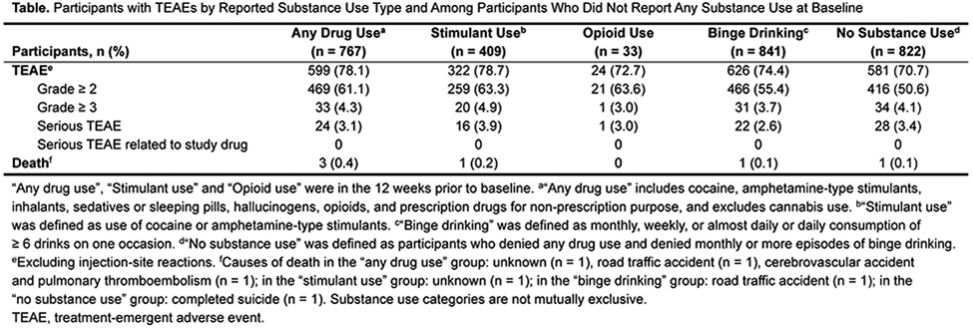

**Methods:**

Substance use was defined as any self-reported drug use, stimulant use, and opioid use in the 12 weeks prior to baseline, and/or binge drinking (≥ 6 drinks on 1 occasion). Adherence (on-time injections) and safety (adverse events [AEs]) through the primary analysis were compared across participants reporting different types of substance use and those reporting no substance use. Prior *in vitro*, clinical, and published fentanyl PK data were used to develop a PBPK model to evaluate the impact of LEN on fentanyl concentrations.

**Results:**

Of 2183 participants randomized to LEN, 767/2061 (37.2%) reported any drug use, 17/2092 (0.8%) injected drugs, 409/2058 (19.9%) used stimulants, 33/2086 (1.6%) used opioids, and 841/2074 (40.5%) had monthly or more binge-drinking episodes at baseline. LEN adherence was high and comparable across substance use types and to participants who did not report substance use (Figure). No substance use–related overdoses or study drug-related serious AEs were reported, and overall incidence of AEs was similar across groups (Table). The most common AEs (excluding injection-site reactions) were gonococcal, chlamydia, and upper respiratory tract infections. PBPK modeling indicated no clinically significant elevations in fentanyl PK with concomitant LEN.

**Conclusion:**

In PURPOSE 2, a population with high rates of substance use, there was high adherence to twice-yearly SC LEN for PrEP and a favorable safety profile among participants who used drugs and alcohol. There were no substance use–related overdoses and no clinically significant impact of LEN on fentanyl concentrations was found. These data support twice-yearly SC LEN to prevent HIV in people who use substances.

**Disclosures:**

Allison Agwu, MD, ScM, Gilead Biosciences: Grant/Research Support|Gilead Biosciences: Site PI of multi-site project & investigator-initiated grant (funds to institution) Jill Blumenthal, MD MAS, Clinical Care Options: speaking fees|Gilead Biosciences: Advisor/Consultant|Gilead Biosciences: Grant/Research Support

